# Responses in Zinc Uptake of Different Mycorrhizal and Non-mycorrhizal Crops to Varied Levels of Phosphorus and Zinc Applications

**DOI:** 10.3389/fpls.2020.606472

**Published:** 2020-12-03

**Authors:** Bao-Gang Yu, Xiu-Xiu Chen, Wen-Qing Cao, Yu-Min Liu, Chun-Qin Zou

**Affiliations:** College of Resources and Environmental Sciences, National Academy of Agriculture Green Development, Key Laboratory of Plant-Soil Interactions, Ministry of Education, China Agricultural University, Beijing, China

**Keywords:** phosphorus, zinc uptake, mycorrhizal and non-mycorrhizal crops, maize, soybean, oilseed rape

## Abstract

Negative effects of high phosphorus (P) application on zinc (Zn) nutrition have been observed in many crops. This study investigated the Zn responses of three typical crops to varied P and Zn applications. A pot experiment was conducted using two mycorrhizal crops (maize and soybean) and one non-mycorrhizal crop (oilseed rape) under three levels of P, two levels of Zn, and two levels of benomyl. Results showed that P application significantly decreased shoot and root Zn concentrations, Zn uptake, and Zn acquisition efficiency (ZnAE) of the three crops irrespective of Zn rate, and that these reductions were greater for maize and soybean than for oilseed rape. Zn application alleviated the P inhibition of Zn uptake in the three crops. The arbuscular mycorrhizal fungi (AMF) colonization of maize and soybean contributed most to the negative effects of increasing P application on Zn uptake, explaining 79–89 and 64–69% of the effect, respectively. For oilseed rape, root dry weight and root Zn concentration explained 90% of the decrease in Zn uptake caused by P application. These results suggest that there is another pathway in addition to the mycorrhizal pathway regulating Zn uptake under mediation by P supply.

## Introduction

Zinc (Zn) is an essential micronutrient in plant growth and development and also plays a vital role in protecting the health of the brain and nervous system of infants and children (Cakmak, 2008). Almost 50% of cereal crops are cultivated in Zn-deficient soil, which contributes to plant and human Zn malnutrition (Zou et al., 2012; Shahzad et al., 2014). According to a report from the WHO (2016), Zn deficiency affects one-third of people worldwide and is particularly prevalent in developing countries.

High application of phosphorus (P) fertilizer is the main reason for low Zn concentration in cereal crops. Many studies have shown that Zn concentration in tissue and grain is significantly decreased by P application (Zhang et al., 2012; Chen et al., 2017). The complex negative interplay between P and Zn has drawn much attention since the 1960s, but there have been no convincing explanations of the mechanism of this antagonism until recently (Stukenholtz et al., 1966; Bouain et al., 2014b).

The negative effects of higher P application on Zn concentration in grain mainly occur in the following processes: uptake, translocation, and remobilization. Zhang et al. (2015, 2017a, b) and Zhang W. et al. (2016) demonstrated that P application significantly affected root uptake of Zn, whereas Zn translocation from roots to shoots and remobilization efficiency were not consistently affected. The significant decrease in Zn uptake is the key factor explaining the antagonistic interaction between P and Zn (Singh et al., 1986; Yang et al., 2011). Nonetheless, understanding of how P application influences root uptake of Zn is limited.

Arbuscular mycorrhizal fungi (AMF) can form a mutualistic relationship with 80% of terrestrial plants (including most crops), in which the extensive extraradical mycelium in the soil enhances immobile nutrient (such as P and Zn) uptake for the host plant in exchange for photosynthesis products from the plant for the fungus (Smith et al., 2003; Cavagnaro, 2008). A meta-analysis by Lehmann et al. (2014) showed that AMF had positive effects on Zn concentration in fruit (grain), shoot, and root of plants. A field experiment conducted on calcareous soils indicated that Zn uptake of wheat increased linearly with increases in AMF colonization (Hui et al., 2019). However, because AMF colonization is negatively correlated with P application, the quantity of Zn taken by AMF can be greatly reduced by P application (Zhang et al., 2017a). A pot and hydroponics experiment suggested that the only reason for the reduction in tissue Zn concentration of wheat caused by P application was the reduction of AMF colonization (Ova et al., 2015). However, Watts-Williams et al. (2013) conducted a pot experiment with mycorrhiza-defective tomato (*rmc*) and its wild-type progenitors (76R) and found that Zn concentration in shoots and roots of *rmc* and 76R was significantly decreased by P application. A solution culture experiment (i.e., mycorrhiza-defective environment) also showed that root Zn uptake of lettuce significantly decreased with increasing P concentration in the nutrient solution (Bouain et al., 2014a). That is, the negative effects of higher P application on Zn uptake can be partially explained by the AMF pathway. Overall, the mycorrhizal pathway has been considered a vital factor explaining the antagonistic interaction between P and Zn. However, the contribution of AMF colonization to the decrease in Zn uptake caused by P application among mycorrhizal crops remains unclear.

Plant species with different root morphologies or physiological characteristics can respond differently to AMF (Tawaraya, 2003). Tran et al. (2019) examined the responses of 15 plant species in pots and found large variation in crop growth and nutritional responses to AMF, i.e., growth and Zn nutrition responses vary among different AMF colonization plants. Moreover, a meta-analysis conducted by Hoeksema et al. (2010) suggested that C_4_ grasses (e.g., maize) respond more positively to AMF inoculation than C_3_ grasses (e.g., soybean). To the best of our knowledge, many studies have focused primarily on the responses of different plant species, crop genotype growth, or a single type of nutrition to AMF, but the negative effects of higher P application on Zn concentration and Zn uptake of different mycorrhizal and non-mycorrhizal crops under the same growth conditions, which is very important for elucidating the Zn uptake pathway mediated by P application, have not yet been reported.

As representatives of main cereal crops, legume crops, and oil-producing crops, maize (*Zea mays* L.), soybean (*Glycine max* L.), and oilseed rape (*Brassica napus* L.) are important dietary sources of calories, proteins, and micronutrients. Thus, it is valuable to clarify the mechanisms of Zn uptake as mediated by P application of these three crops. Among these crops, both maize and soybean are highly mycorrhizal crops under current agricultural management practices, but oilseed rape is considered a non-mycorrhizal crop (Buee et al., 2000; Chu et al., 2020; Qin et al., 2020). Besides, as a general fungicide, benomyl can effectively suppress AMF colonization, in which it is considered as an ideal method to evaluate mycorrhizal responsiveness (Yang et al., 2014; Qiao et al., 2019). Therefore, we selected two typical mycorrhizal crops, maize and soybean, and one non-mycorrhizal crop, oilseed rape, to study the different responses of Zn concentration and Zn uptake to various levels of P, Zn, and benomyl application and to clarify the contributions of potential factors to the antagonistic interaction of P and Zn. We hypothesized that P application significantly decreased Zn uptake of mycorrhizal and non-mycorrhizal crops, the decreases in Zn uptake of mycorrhizal crops caused by P application were mainly attributed to AMF colonization decline, and the decrease of non-mycorrhizal crops was mainly due to root system architecture.

## Materials and Methods

### Experimental Design

A pot experiment was conducted from March to May 2018 at China Agriculture University (CAU). The soil used in this experiment was from the Quzhou Experiment Station (36.9°N, 115.0°E) of CAU. The initial soil pH was 8.01, and soil available Zn (DTPA-Zn) and P concentrations (Olsen-P) were 0.93 and 9.97 mg kg^–1^, respectively (The corresponding analysis methods are shown in section “Harvesting and Analyses”). Two Zn levels (0 and 30 mg Zn kg^–1^ soil) as ZnSO_4_⋅7H_2_O and three P levels (0, 200, and 600 mg P kg^–1^ soil) in the form of Ca(H_2_PO_4_)_2_ were applied in this experiment. To establish different AMF colonization, two benomyl levels (0 and 0.4 g kg^–1^ soil) were used based on a previous study (Fitter and Nichols, 1988). In addition, 200 mg nitrogen (N) kg^–1^ soil in the form of urea and 200 mg K_2_O kg^–1^ soil in the form of K_2_SO_4_ were applied uniformly to all treatments before sowing. Benomyl was dissolved in 1 L distilled water and total irrigated four times (once every 2-week period). An equivalent amount of distilled water was irrigated in no-benomyl-addition treatments to create a similar soil moisture condition. Each pot contained 3.5 kg soil. All crops were arranged in a glasshouse with a randomized complete block design with three replicates and were watered with distilled water as needed.

Maize (*Z. mays* L., cv. Zhengdan 958), soybean (*G. max* L., cv. Zhonghuang 37), and oilseed rape (*B. napus* L., cv. Meiyou 758) seeds were planted in this experiment. Five germinated maize and soybean seeds and eight germinated oilseed rape seeds were transferred to pots after germination. At the three-leaf stage of maize, each pot was thinned to three plants. Soybean and oilseed rape were thinned to two and five plants per pot within 1 week after sowing, respectively.

### Harvesting and Analyses

Seven weeks after sowing, the shoots and roots of each plant were harvested. Shoots were cut off at ground level using stainless steel scissors. All shoot and root samples were washed three times with tap water and distilled water, respectively. Fresh root sub-samples with a mass of 0.5 g were used to evaluate arbuscular mycorrhizal colonization. Segments with a length of 1 cm were soaked in KOH (10%), HCl (2%), trypan blue (0.05%), and acidic glycerol, respectively (Koske and Gemma, 1989). Then, arbuscular mycorrhizal colonization was quantified using the magnified intersections method (McGonigle et al., 1990).

Shoots and remaining roots were oven-dried at 60–65°C to constant weight to record dry weight and then were ground with a stainless-steel grinder for Zn nutrition analyses. The plant samples were digested with HNO_3_–H_2_O_2_ using a microwave-accelerated reaction system (CEM, Matthews, NC, United States), and the Zn concentration in the digestate was analyzed by inductively coupled plasma optical emission spectroscopy (ICP-OES; OPTIMA 3300 DV; Perkin-Elmer, United States). Standard materials (IPE126) from Wageningen Evaluation Programs for Analytical Laboratories (WEPAL; Wageningen University, Netherlands) were used to check the Zn concentrations in shoots and roots.

Soil samples were collected, air-dried, and then passed through a 1 mm plastic sieve for analyses of available Zn and P concentrations. The available Zn concentration was extracted using 5 mmol L^–1^ diethylenetriaminepentaacetic acid (DTPA) and analyzed by ICP-OES (OPTIMA 3300 DV; Perkin-Elmer, United States) (Lindsay and Norvell, 1978). The soil available P concentration was extracted using 0.5 mol L^–1^ NaHCO_3_ and analyzed using a spectrophotometer (Olsen, 1954). Soil pH (1:2.5 w/v in water) was determined by a pH meter (PB-10; Sartorius, Germany).

### Calculation and Statistical Analyses

Zn acquisition efficiency (ZnAE) was calculated according to Zhu et al. (2001):

ZnAE = total Zn accumulation/root dry weight.

For further analyses of the effects of AMF on Zn nutrition, mycorrhizal Zn responsiveness (MZnR) was calculated according to Gao et al. (2007):

MZnR = ([Zn accumulation_*B*__0_ – Zn accumulation_*B*__0_._4_]/Zn accumulation_*B*__0_._4_) × 100

where Zn accumulation_*B*__0_._4_ and Zn accumulation_*B*__0_ represent the total Zn accumulation of crops treated with and without benomyl addition, respectively.

Three-way analyses of variance (ANOVAs) were conducted to test the main effects of P, Zn, and benomyl additions and their interactions on the dry weights of shoots and roots, Zn concentration in shoots and roots, total Zn accumulation, ZnAE, and AMF colonization. Similarly, two-way ANOVAs were carried out to test the main effects of P and Zn applications and their interactions on MZnR. Following ANOVA, means for different P levels were compared within Zn and benomyl application levels using SAS software (version 8.0). SPSS software (version 26.0) was used to perform path coefficient analyses to evaluate the contributions of potential factors to the antagonistic interaction between P and Zn.

## Results

### Shoot and Root Dry Weights

P and Zn applications significantly increased shoot and root dry weights of maize, soybean, and oilseed rape. Benomyl addition significantly decreased shoot and root dry weights of maize and soybean but not of oilseed rape. There were significant interactions between P and Zn application levels for the three crops (Table 1). When no Zn was applied, irrespective of the level of benomyl addition, shoot and root dry weights of maize, soybean, and oilseed rape increased with P application from 0 to 200 mg kg^–1^ and then decreased with further P application to 600 mg kg^–1^ (Table 1). When Zn was applied at 30 mg kg^–1^, P application (200 and 600 mg kg^–1^) led to 24–42, 22–41, and 20–33% increases in the shoot dry weights of maize, soybean, and oilseed rape compared with no P application, respectively, when no benomyl was added. There were similar increases in shoot and root dry weights when 30 mg Zn kg^–1^ and 0.4 g kg^–1^ benomyl was added (Table 1).

**TABLE 1:** Shoot and root dry weights of maize, soybean, and oilseed rape affected by P application (P), Zn application (Zn), and benomyl addition (B), respectively.

B level (g kg^–1^)	Zn level (mg kg^–1^)	P level (mg kg^–1^)	Shoot dry weight (g plant^–1^)			Root dry weight (g plant^–1^)		
			Maize	Soybean	Oilseed rape	Maize	Soybean	Oilseed rape
0	0	0	4.3b	2.8b	2.3b	0.54b	0.35b	0.29b
		200	5.4a	3.4a	2.7a	0.63a	0.40a	0.32ab
		600	5.1ab	3.1a	2.3ab	0.56ab	0.39ab	0.30b
	30	0	4.6c	2.9c	2.4b	0.56b	0.36b	0.30b
		200	5.7b	3.6b	2.9a	0.69ab	0.43a	0.35a
		600	6.5a	4.1a	3.2a	0.72a	0.44a	0.38a
0.4	0	0	3.4b	2.6b	2.3b	0.36b	0.29b	0.27b
		200	4.7a	3.0a	2.5a	0.44a	0.34a	0.30a
		600	4.6a	2.8ab	2.5ab	0.41ab	0.31ab	0.28b
	30	0	3.7c	2.8c	2.4b	0.40c	0.30b	0.31b
		200	5.0b	3.1b	2.9a	0.54b	0.36ab	0.32ab
		600	6.0a	3.5a	3.0a	0.65a	0.38a	0.34a
Source of variation								
P level (P)			***	***	***	***	***	**
Zn level (Zn)			***	***	***	***	***	***
B level (B)			***	***	ns	***	***	ns
P × Zn			**	***	***	**	*	**
P × B			ns	**	ns	ns	ns	ns
Zn × B			ns	ns	ns	ns	ns	ns
P × Zn × B			ns	ns	ns	ns	ns	ns

Values are means of three replications. For each benomyl and Zn treatment, means with the same lowercase letter indicate no significant difference among P application levels (*P* < 0.05). *, **, and *** significance at *P* < 0.05, 0.01, and 0.001, respectively. ns, no significance, *P* > 0.05.

### Zn Concentration, Accumulation, and Acquisition Efficiency

**FIGURE 1:**
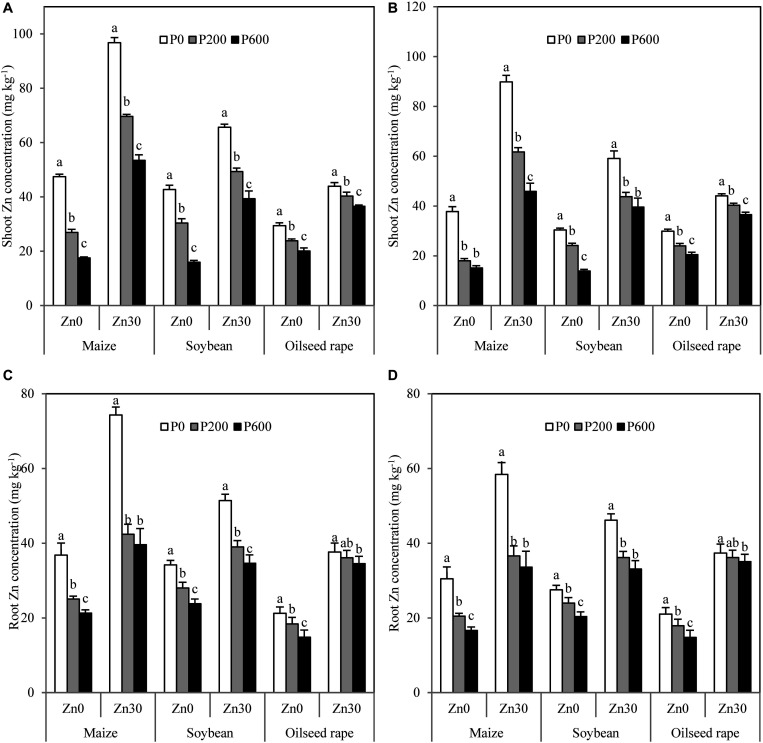
Zn concentration in shoots and roots of maize, soybean, and oilseed rape affected by P and Zn applications without benomyl **(A,C)** and with 0.4 g benomyl kg^–1^ soil **(B,D)** addition, respectively. Values are means of three replications. The same lowercase letter indicates no significant difference among P application levels (*P* < 0.05). Zn0 and Zn30 represent 0 and 30 mg Zn kg^–1^ soil rates, respectively. P0, P200, and P600 represent 0, 200, and 600 mg P kg^–1^ soil rates, respectively.

**FIGURE 2:**
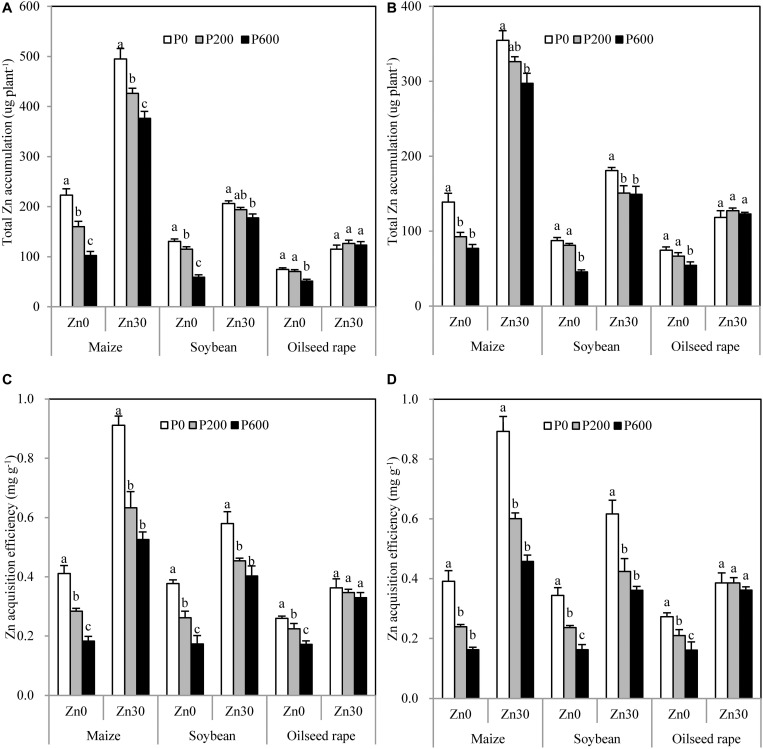
Total Zn accumulation and Zn acquisition efficiency of maize, soybean, and oilseed rape affected by P and Zn applications without benomyl **(A,C)** and with 0.4 g benomyl kg^–1^ soil **(B,D)** addition, respectively. Values are means of three replications. The same lowercase letter indicates no significant difference among P application levels (*P* < 0.05). Zn0 and Zn30 represent 0 and 30 mg Zn kg^–1^ soil rates, respectively. P0, P200, and P600 represent 0, 200, and 600 mg P kg^–1^ soil rates, respectively.

Zn concentration in shoots and roots was significantly decreased in all crops by P application regardless of Zn and benomyl additions (Figure 1). When no Zn was applied, total Zn accumulation (Figures 2A,B) and ZnAE (Figures 2C,D) were significantly decreased in all crops by P application, and these traits in maize and soybean but not in oilseed rape were also significantly decreased by P application when 30 mg Zn kg^–1^ was added (Figure 2). Benomyl addition also decreased Zn concentration in shoots and roots, total Zn accumulation, and ZnAE in maize and soybean but not in oilseed rape (Table 2). Irrespective of P and benomyl additions, Zn application obviously increased these three traits in all crops (Table 2). Compared with no P application, P application led to 43–63, 29–60, and 19–31% decreases in Zn concentration in shoots of maize, soybean, and oilseed rape when no Zn or benomyl was added (Figure 1A). There were similar decreases in Zn concentration in the roots of all three crops (Figures 1C,D). Meanwhile, 600 mg P kg^–1^ application decreased the total Zn accumulation of maize, soybean, and oilseed rape by 55, 54, and 31% compared with no P application when no Zn or benomyl was added, respectively. When 30 mg Zn kg^–1^ was added but no benomyl, the total Zn accumulation of maize and soybean decreased by 24 and 14% due to application of 600 mg P kg^–1^ (Figures 2A,B). The ZnAEs of maize, soybean, and oilseed rape without Zn or benomyl addition were reduced by 32–56, 31–52, and 15–35% by 200 and 600 mg P kg^–1^ applications, respectively (Figures 2C,D). More interestingly, the ZnAE of maize was higher than that of soybean, followed by oilseed rape (Figures 2C,D).

**TABLE 2:** Analysis of variance for the effects of P application (P), Zn application (Zn), benomyl addition (B), and their interactions on Zn concentration in shoots and roots, total Zn accumulation, Zn acquisition efficiency, and arbuscular mycorrhizal fungi (AMF) colonization of maize, soybean, and oilseed rape, respectively.

Source	Shoot Zn concentration			Root Zn concentration			Total Zn accumulation			Zn acquisition efficiency			AMF colonization	
	Maize	Soybean	Oilseed rape	Maize	Soybean	Oilseed rape	Maize	Soybean	Oilseed rape	Maize	Soybean	Oilseed rape	Maize	Soybean
P level (P)	***	***	***	***	***	***	***	***	**	***	***	***	***	***
Zn level (Zn)	***	***	***	***	***	***	***	***	***	***	***	***	***	***
B level (B)	***	***	ns	***	***	ns	***	***	ns	*	**	ns	***	***
P × Zn	***	*	*	***	***	**	*	***	***	***	*	ns	*	***
P × B	ns	**	ns	ns	**	ns	***	ns	ns	ns	ns	ns	***	***
Zn × B	ns	ns	ns	ns	ns	ns	***	ns	ns	ns	ns	ns	**	***
P × Zn × B	ns	ns	ns	ns	ns	ns	ns	ns	ns	ns	ns	ns	**	*

*, **, and *** significance at P < 0.05, 0.01, and 0.001, respectively. ns, no significance, P > 0.05.

### AMF Colonization and MZnR

**FIGURE 3:**
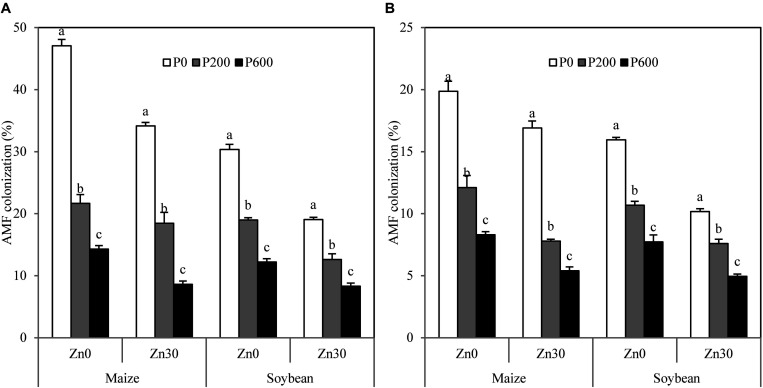
Arbuscular mycorrhizal fungi (AMF) colonization of maize and soybean affected by P and Zn applications without benomyl **(A)** and with 0.4 g benomyl kg^–1^ soil **(B)** addition, respectively. Values are means of three replications. The same lowercase letter indicates no significant difference among P application levels (*P* < 0.05). Zn0 and Zn30 represent 0 and 30 mg Zn kg^–1^ soil rates, respectively. P0, P200, and P600 represent 0, 200, and 600 mg P kg^–1^ soil rates, respectively.

No AMF colonization was detected in root segments of oilseed rape in any treatment (data not shown). The addition of P, Zn, and benomyl significantly decreased AMF colonization of maize and soybean (Table 2). There were significant (Table 2) three-way interactions among P, Zn, and benomyl treatments of both maize and soybean. Compared with no P application, P application led to 54–70 and 37–60% decreases in AMF colonization of maize and soybean when no Zn or benomyl was added, respectively. The same decreases were observed in maize and soybean at other Zn and benomyl addition levels (Figure 3). As there were no significant differences in total Zn accumulation of oilseed rape between the no benomyl and the 0.4 g kg^–1^ benomyl treatments (Table 2), only the MZnR values of maize and soybean were calculated. P and Zn applications significantly decreased the MZnRs of three crops (Table 3). P application decreased that of maize by 45% when no Zn was applied and by 36% when 30 mg Zn kg^–1^ was applied. When no Zn was applied, P application significantly decreased the MZnR of soybean by 40%, but there were no significant differences for the 30 mg Zn kg^–1^ treatment (Table 3). In addition, AMF colonization and MZnR were consistently higher in maize than in soybean (Figure 3 and Table 3).

**TABLE 3:** Mycorrhizal Zn responsiveness of maize and soybean affected by P and Zn applications, respectively.

		Mycorrhizal Zn responsiveness (%)	
Zn level (mg kg^–1^)	P level (mg kg^–1^)	Maize	Soybean
0	0	61.2a	50.1a
	200	68.4a	42.2ab
	600	33.5b	30.2b
30	0	40.0a	18.8a
	200	30.7ab	21.0a
	600	26.6b	20.3a
Source of variation			
P level (P)		***	**
Zn level (Zn)		***	*
P × Zn		**	*

Values are means of three replications. Means with the same lowercase letter indicate no significant difference among P application levels (P < 0.05). *, **, and *** significance at P < 0.05, 0.01, and 0.001, respectively.

### Correlations Between Total Zn Accumulation and Root Zn Concentration, Root Dry Weight, and AMF Colonization

We selected total Zn accumulation as the dependent variable and seven independent variables (shoot dry weight, root dry weight, shoot Zn concentration, root Zn concentration, AMF colonization, soil DTPA-Zn concentration, and soil Olsen-P concentration) to uncover how and to what extent the independent variables influenced the total Zn accumulation of each crop; however, only root dry weight, root Zn concentration, and AMF colonization entered into the analyses process for the three crops (Table 4). Overall, when no Zn was applied, AMF colonization explained 89% of the total Zn accumulation of maize; AMF colonization, root dry weight, and root Zn concentration explained 90% of the total accumulation of soybean; and root dry weight and root Zn concentration explained 90% of the total accumulation of oilseed rape. When Zn was applied at 30 mg kg^–1^, AMF colonization and root dry weight explained 88% of the total accumulation of maize, AMF colonization explained 64% of the total accumulation of soybean, and root dry weight and root Zn concentration explained 92% of the total accumulation of oilseed rape (Table 4).

**TABLE 4:** Correlations of total Zn accumulation (dependent variable) with independent variables [root Zn concentration (RZnC), root dry weight (RDW), and arbuscular mycorrhizal fungi colonization (AMFC)] for maize, soybean, and oilseed rape when Zn was applied at 0 or 30 mg kg^–1^ soil.

							Indirect path coefficients		
Zn level (mg kg^–1^)	Crop	Dependent variable	Independent variable	Correlation coefficient	Adjusted *R*^2^	Path coefficient			
							AMFC	RDW	RZnC
0	Maize	Total Zn accumulation	AMFC	0.95	0.89	0.95	–		
	Soybean	Total Zn accumulation	AMFC	0.84	0.69	0.17	–	0.09	0.59
			RDW	0.64	0.76	0.55	0.03	–	0.06
			RZnC	0.82	0.90	0.61	0.16	0.05	–
	Oilseed rape	Total Zn accumulation	RDW	0.87	0.75	0.95		−0.07	–
			RZnC	0.35	0.90	0.49		–	−0.14
30	Maize	Total Zn accumulation	AMFC	0.90	0.79	0.96	–	−0.06	
			RDW	0.10	0.88	0.31	−0.20	–	
	Soybean	Total Zn accumulation	AMFC	0.80	0.64	0.80	–		
	Oilseed rape	Total Zn accumulation	RDW	0.01	0.79	1.11		−0.21	–
			RZnC	0.89	0.92	0.49		–	−0.48

## Discussion

### Effect of P Application on Zn Concentration and Uptake

P application significantly decreased the Zn concentration of both maize and soybean, consistent with previous reports (Izsáki, 2014; Zhang et al., 2017a). It is worth noting that significant decreases in Zn concentration in shoots and roots with increasing P application were also observed in non-mycorrhizal crop, oilseed rape (Figure 1). This is consistent with previous research that showed that Zn concentrations in crambe (*Crambe abyssinica* Hochst, i.e., a non-mycorrhizal crop) significantly decreased with increasing P application (Cihacek et al., 1993). Considering the large changes in biomass in response to P application, the responses of total Zn accumulation due to P application were partially different from those of Zn concentration, in line with Chen et al. (2019). When no Zn was applied, total Zn accumulations were significantly decreased by P application in all crops. The negative effects of increasing P application on Zn accumulation in oilseed rape are not consistent with Lu et al. (1998), who found that a 150 mg P kg^–1^ application increased shoot and root Zn accumulations in two oilseed rape genotypes. The reason for the disagreement may be the relatively lower P fertilizer application they used than that used in the present study. Similarly, the results of a field experiment showed that the total Zn accumulation of wheat was increased by insufficient P application but decreased by excessive P application (Zhang W. et al., 2016). Together, these results suggest that the effects of P on Zn uptake depend on the amount of P supplied. P application also decreased the total Zn accumulation of maize and soybean but not of oilseed rape when 30 mg Zn kg^–1^ was applied, and the degree of decrease was lower at 30 mg Zn kg^–1^ than under no Zn addition for all crops, indicating that reasonable Zn fertilization can alleviate the inhibition of P application on crop Zn uptake.

Our results indicate that chemical interaction in soil was not the reason for the decrease in Zn uptake caused by P application because the soil Olsen-P concentration did not reach the critical value of 200 mg kg^–1^ (Chen et al., 2019). P application significantly increased soil DTPA-Zn concentration of maize and soybean as Zn applied, which is consistent with Saeed (1977) and Zhang et al. (2017a). However, no significant effect of P application on soil DTPA-Zn concentration was found for oilseed rape (Supplementary Figures 1C,D). Our results showed that P application had no significant effects on soil pH of three crops (Supplementary Figures 1A,B). Therefore, the differences in DTPA-Zn between mycorrhizal and non-mycorrhizal crops are possibly ascribed to the mobilized soil Zn induced by AMF hyphal exudates with P application (Kaiser et al., 2015; Zhang L. et al., 2016). Previous studies have ascribed the decrease in Zn concentration with P application to a “dilution effect,” in which concentration decreases with biomass increase (Hui et al., 2019). However, in the current study, the shoot and root dry weights of maize, soybean, and oilseed rape did not increase as P application increased to 600 mg kg^–1^, in line with previous studies (Li et al., 2003; Ova et al., 2015). These results indicate that the decrease in Zn concentration in shoots and roots of the three crops caused by P application cannot simply be ascribed to the dilution effect. This was further demonstrated by the significant decrease in ZnAE of the three crops with increasing P application (Figures 2C,D), which also agrees with previous findings (Zhu et al., 2001; Zhang W. et al., 2016). Taken together, these results provide specific evidence that decreased uptake of Zn is a vital process in the antagonism of P and Zn. However, the process of Zn uptake in the rhizosphere may be affected by AMF colonization, root growth, and root Zn concentration (Zhang et al., 2017a).

### Different Zn Uptake Responses Caused by P Application in Different Test Crops

Although the responses of different crop species and genotypes to P or Zn application have been verified to be different (Yilmaz et al., 1997; Fageria, 2002; Watts-Williams et al., 2019; Qin et al., 2020), the negative effects of increasing P application on Zn uptake among different mycorrhizal and non-mycorrhizal crops have not been reported. In the present study, mycorrhizal crops (maize and soybean) displayed higher degrees of decrease in Zn uptake and ZnAE mediated by P application than non-mycorrhizal crop (oilseed rape). The main reason for this difference may be that P application sharply decreased AMF colonization of maize and soybean, which reduced the quantity of Zn uptake by the mycorrhizal pathway. Analogous results were reported by Watts-Williams and Cavagnaro (2012), who found that the degree of decrease in Zn concentration caused by P application was greater in 76R (mycorrhizal genotypes of tomato) than in *rmc* (non-mycorrhizal genotypes of tomato). In our study, between the two mycorrhizal crops, maize displayed greater decreases in Zn uptake and ZnAE in response to P application than soybean. This was related to AMF colonization, and the MZnR of maize was consistently higher than that of soybean (Figure 3 and Table 3).

Our path coefficient analysis results quantified the factors and the extents of influence on the Zn uptake of three crops caused by P application. Consistent with the results of total Zn accumulation and ZnAE, the contributions of possible factors to the decrease in Zn uptake attributable to P application depended on whether Zn fertilizer was applied to the soil. AMF colonization of maize and soybean contributed most to the negative effects of higher P application on Zn uptake, explaining 89 and 69% of the effects when no Zn was applied and 79 and 64% at 30 mg Zn kg^–1^, respectively (Table 4). The decrease in the contribution of AMF colonization at 30 mg Zn kg^–1^ may be attributable to significantly decreased AMF colonization under Zn fertilization (Figure 3). Our results further demonstrate that the mycorrhizal pathway is an important factor explaining the negative effects of increasing P application on the Zn uptake of mycorrhizal crops, and that more mycorrhizal crops are affected by AMF colonization as for the antagonism of P on Zn. For oilseed rape, root dry weight and root Zn concentration explained 90 and 92% of the decrease in Zn uptake at the no Zn and 30 mg Zn kg^–1^ application levels, respectively. This indicates that root growth and development are vital factors explaining the antagonism of non-mycorrhizal crops. In addition, our results revealed that 11–12, 10–36, and 8–9% of the decreases in Zn uptake of maize, soybean, and oilseed rape caused by P application, respectively, could not be explained in the present study. This implies that there are other pathways regulating Zn uptake as mediated by P application irrespective of whether the crop is mycorrhizal or non-mycorrhizal.

This study strengthens understanding of the different responses of the negative effects of higher P application on Zn concentration and Zn uptake of different mycorrhizal and non-mycorrhizal crops and further highlights the importance of AMF colonization in this process. Other pathways pertaining to the Zn uptake mediated by P application need to be considered in future research. In the process of crop development, different root system architectures can form due to the soil environment, which may directly influence the nutrient acquisition capacity (Xue et al., 2014). Moreover, some Zn transporters play important roles in root uptake of Zn, and whether this is mediated by P supply remains unclear. Future studies need to investigate whether and how P application affects root system architecture and the expressions of genes related to Zn uptake and root Zn uptake. Besides, though this study clarified the contribution of AMF colonization to the antagonistic interaction of P and Zn among mycorrhizal crops, there are some important points to be considered in the future. For instance, in the current study, we selected benomyl to establish different levels of AMF colonization, but it may affect various members of the soil microbial community and thus potentially influence the growth and development of crops in other ways (Rillig, 2004). Using a mycorrhiza-defective mutant is a possibility for comparing mycorrhizal and non-mycorrhizal crops in native soil without any other experimental intervention (Watts-Williams and Cavagnaro, 2015). Thus, there may be theoretical significance in quantifying the contribution of AMF colonization in this antagonistic interaction using mycorrhiza-defective mutants and their wild-type genotypes.

## Conclusion

Three typical crops showed different responses in growth and Zn uptake to P application levels. P application significantly decreased the Zn uptake of both mycorrhizal and non-mycorrhizal crops, and the degree of reduction depended on whether the crop is mycorrhizal or non-mycorrhizal and the Zn supply. The decrease in Zn uptake of maize and soybean was mainly due to the decreased AMF colonization caused by P application, whereas root dry weight was significant for oilseed rape. Our results indicate that there is another pathway regulating Zn uptake mediated by P supply in addition to the mycorrhizal pathway.

## Data Availability Statement

The raw data supporting the conclusions of this article will be made available by the authors, without undue reservation.

## Author Contributions

B-GY contributed to resources, data curation, and writing of the original draft. X-XC contributed to investigation. W-QC contributed to investigation. Y-ML contributed to investigation. C-QZ contributed to conceptualization, writing, review, and editing. All authors contributed to the article and approved the submitted version.

## Conflict of Interest

The authors declare that the research was conducted in the absence of any commercial or financial relationships that could be construed as a potential conflict of interest.
