# Supplementary material for: Responses in Zinc Uptake of Different Mycorrhizal and Non-mycorrhizal Crops to Varied Levels of Phosphorus and Zinc Applications
Source: Front Plant Sci. 2020 Dec 3;11:606472. doi: 10.3389/fpls.2020.606472 (PMC7744350; doi:10.3389/fpls.2020.606472)
Supplement: Supplementary file 1 [file Table_1.DOCX]

***Supplementary Material***

**(B)**

**(A)**

**(D)**

**(C)**

**(F)**

**(E)**

**Supplementary Fig. 1** Soil pH, DTPA-Zn, and Olsen-P concentration after harvesting under P and Zn application without benomyl (A, C, and E) and with 0.4 g benomyl kg^–1^ soil (B, D, and F) addition, respectively. Values are means of three replications. The same lowercase letter indicates no significant difference among P application levels (*P* < 0.05). Zn0 and Zn30 represent 0 and 30 mg Zn kg^–1^ soil rates, respectively. P0, P200, and P600 represent 0, 200, and 600 mg P kg^–1^ soil rates, respectively.
